# Housekeeping Genes for Parkinson’s Disease in Humans and Mice

**DOI:** 10.3390/cells10092252

**Published:** 2021-08-30

**Authors:** Anelya Kh. Alieva, Elena V. Filatova, Margarita M. Rudenok, Petr A. Slominsky, Maria I. Shadrina

**Affiliations:** Institute of Molecular Genetics of National Research Centre “Kurchatov Institute”, 2 Kurchatova Sq., 123182 Moscow, Russia; FilatovaEV@img.ras.ru (E.V.F.); margaritamrudenok@gmail.com (M.M.R.); slomin@img.ras.ru (P.A.S.); shadrina@img.ras.ru (M.I.S.)

**Keywords:** reference gene, housekeeping gene, gene expression, neurodegeneration, Parkinson’s disease, real-time PCR, TaqMan

## Abstract

A critical aspect of real-time PCR is the presence of housekeeping genes (HKGs) as an internal control for the normalization of expression data for genes of interest. It is necessary to select correct HKGs in the investigation of various pathologies. Thereby, we analyzed the stability of expression of the HKGs in Parkinson’s disease (PD). The work was carried out in the peripheral blood of patients with PD and in the brain tissues and peripheral blood of mice with MPTP-induced PD. As a result, *Aars* was the most stably expressed HKG in the mouse brain as a whole. However, different genes were more stably expressed in different parts of the brain. *Polr2f* was the most stably expressed in the cortex, *Psmd6* was the most stably expressed in the cerebellum, and *Psmd7* was the most stably expressed in the striatum and substantia nigra. HKGs were different in similar tissues of the studied organisms. *Polr2f* was the most stably expressed HKG in the peripheral blood of mice, whereas *PSMD6* was the most stably expressed gene in humans. Thus, there is no universal HKG both for different brain tissues of one organism and for similar tissues of different organisms. Furthermore, the identified most stably expressed HKGs can be considered as such only under conditions in PD.

## 1. Introduction

Currently, active investigations of gene expression are being carried out. The simplest and most widely used method for analysis of gene expression at the mRNA level is a semiquantitative approach based on the reverse transcription (RT) reaction and real-time quantitative polymerase chain reaction (qPCR). This method is considered the gold standard for the study of changes in gene expression at the mRNA level in biomedical investigations [1,2].

A critical aspect of this method, as for other semiquantitative methods, is the presence of reference genes as an internal control for the normalization of expression data for the genes of interest. Based on this, we can conclude that the precise determination of changes in the expression of candidate genes depends on the correct choice of internal reference genes. These genes are called housekeeping genes (HKGs).

Genes with stable expression in all biological samples and whose expression does not change under experimental conditions or under conditions of disease are most often considered as HKGs [3]. HKGs are considered to be genes that control basic metabolism and are responsible for cell survival, maintenance of cell homeostasis, and/or other fundamental processes in the cell [4].

It is believed that the expression of these genes should not depend on the influence of various factors acting on the cell, tissue, and organism, or these factors should have minimal effect. However, it is now becoming clear that the expression of the most common HKGs can depend on external conditions and changes in pathologies [5,6]. The significant disadvantages of using such HKGs in studies are the lack of validation of HKGs in specific experimental conditions, as well as the blind transfer of HKGs selected for one organism to another, without the accompanying careful verification. This, in turn, can leave an imprint on the results obtained and/or lead to false results in assessing changes in the expression of candidate genes [7,8,9]. 

Based on this observation, a search and selection of the most stably expressed HKGs for the analysis of gene expression in Parkinson’s disease (PD) were carried out. PD is one of the most common neurological diseases and is characterized predominantly by the death of dopaminergic neurons in the substantia nigra. Degeneration of dopaminergic neurons leads to disorganization of the function of almost all neurotransmitter systems of the brain. In this regard, the etiopathogenesis of PD has been widely studied, and active analysis of changes in gene expression under conditions of this pathology is being carried out. However, experimental results are often not reproducible or do not match. Perhaps this is due to differences in the HKGs used.

In this regard, we analyzed the reference genes in the peripheral blood of patients with PD, as well as in the tissues of the brain and peripheral blood of mice (Mus musculus), as the most widespread object for modeling PD [10].

## 2. Materials and Methods

### 2.1. Patients

In this study, 35 untreated and 12 treated patients with the sporadic form of PD (stages 1 and 2 of the Hoehn–Yahr scale) were analyzed. The mean age ± standard deviation (SD) at disease onset was 58.6 ± 7.4 years (range 46–71), and the mean age at enrollment was 59.2 ± 7.1 years (range 47–73). The PD patients with treatment received different medications with dopamine receptor agonists (l-dopa, piribedil, pramipexole, or amantadine) in various combinations.

PD patients were diagnosed with PD at the Research Center of Neurology (Moscow, Russia). They were selected and studied according to the International Movement Disorder Society Unified Parkinson’s Disease Rating Scale [11] and Hoehn–Yahr scores [12]. The diagnosis of PD was based on the UK PD Society Brain Bank Criteria [13].

44 neurologically normal healthy individuals were studied as controls. These participants were matched to the PD patients regarding gender, age, and ethnicity.

Patients and neurologically healthy volunteers (of Russian ethnic origin residing in European Russia) were selected at the Research Center of Neurology (Moscow, Russia). All participants were examined for the presence of frequent mutations in all genes responsible for monogenic forms of PD and did not have any family history of PD. All participants had no severe concomitant diseases, such as severe cardiovascular disease, cancer, or diabetes mellitus.

The study was conducted in accordance with the World Medical Assembly Declaration of Helsinki—Ethical Principles for Medical Research Involving Human Subjects. All blood samples were collected with the informed consent of the investigated subjects. The study was approved by the Ethics Committee of the Research Center of Neurology (Moscow, Russia), protocol 18/4.

### 2.2. Mice

Twenty C57BL/6 mice were purchased from the specific pathogen-free licensed nursery of the Shemyakin–Ovchinnikov Institute of Bioorganic Chemistry of the Russian Academy of Sciences. Eight-week-old male mice weighing 22–25 g were randomly divided into two groups for analysis: one control group and one experimental group. Each group of mice contained 10 individual animals. The mice were housed 3–4 to a cage under standard conditions with free access to food and water with a 12:12 h light:dark regime.

### 2.3. Modeling of Parkinson-like Phenotype with MPTP

A mouse model of the early symptomatic stage of PD was used in this study. Experimental work with laboratory animals was carried out in accordance with The Guide for the Care of Animals [14] as approved by the Ethics Committee of the Institute of Molecular Genetics of National Research Centre “Kurchatov Institute” (Moscow, Russia), protocol 3/21. Samples of 5.5 ± 0.35 mg of striatum, cortex, and cerebellum; 1.4 ± 0.22 mg of the substantia nigra; and 477.8 ± 80 μL of peripheral blood were taken from each animal for the study following decapitation.

All efforts were made to minimize the potential suffering and discomfort of animals, and care was taken according to the 3R rule (replacement, reduction, and refinement).

### 2.4. Expression Analysis

#### RNA Isolation

The isolation of total RNA from brain tissues was carried out using the RNAeasy Mini Kit (Qiagen, Germany) and Quick-RNA MiniPrep Kit (Zymo Research Corp., Irvine, CA, USA).

The isolation of total RNA from 250 µL of each sample of human and mouse peripheral blood was performed using Whole Blood RNA MiniPrep Kit (Zymo Research Corp., Irvine, CA, USA). 

The concentration of total isolated RNA from all samples was measured using a Quant-iT RNA BR Assay Kit and a Qubit 3.0 fluorimeter (Life Technologies, Grand Island, NY, USA). RNA quality was monitored using an Experion automated electrophoresis system (Bio-Rad Laboratories, Hercules, CA, USA). The RNA quality index was higher than 8.5 in all samples.

All procedures were carried out according to the manufacturers’ recommendations.

### 2.5. Analysis of Changes in Relative Levels of mRNAs

Gene expression analysis was conducted using RT-qPCR with TaqMan probes. All samples were handled as equally as possible in order to minimize any random biases. Single-stranded DNA was synthesized using 120 ng of total RNA, 100 ng of Escherichia coli tRNA as a carrier [15], specific primers, and the RevertAid H Minus First Strand cDNA Synthesis Kit (Thermo Fischer Scientific Inc., Waltham, MA, USA) according to the manufacturer’s recommendations. Each RT reaction was run in triplicate.

Primers and probes for qPCR were selected using the Beacon Designer 7.0.2 program and the nucleotide sequences of the genes of humans, mice, and rats. The sequences of gene-specific primers and probes are listed in Table S1.

cDNA obtained in the reverse transcription reaction was used as a template for qPCR. This was diluted 50 times in an aqueous solution of tRNA from Escherichia coli (0.02 ng/μL) [15]. qPCR was performed using the StepOnePlus System (Applied Biosystems, USA), QuantStudio 3 System (Thermo Fischer Scientific Inc., Waltham, MA, USA), and PCR reagents (Syntol, Moscow, Russia). Thermal cycling for samples from mice and humans was performed as follows: 60 s at 50 °C; 40 cycles of 15 s at 95 °C and 40 s at 61 °C; 30 s at 25 °C. Thermal cycling for samples from rats was performed as follows: 600 s at 95 °C and 45 cycles: 5 s at 95 °C followed by 10 s at 60 °C. The reaction was repeated three times for each cDNA to correct for differences in sample quality and reverse transcription efficiency.

### 2.6. Statistical Analysis

Primers and probes for analysis were selected using Beacon Designer 7.0 (Premier Biosoft International, San Francisco, CA, USA) and corresponding sequences from the NCBI database [16]. The specificities of primers and probes were checked using Primer3 [17] and Primer-BLAST [18]. Evaluation of expression of reference genes was performed using Cq values and the RefFinder tool [19].

The RefFinder program calculates the score of reference genes using the algorithms of geNorm [7], Normfinder [8], and BestKeeper [20] programs and the comparative ΔΔCt method [21].

Based on these scores, the program assigns an appropriate score to each gene and calculates the geometric mean of their weights for the overall final ranking. The program chooses the lowest score among the ranks assigned by each algorithm separately and then assigns the final comprehensive score. The closer the final score is to one, the more stably expressed the HKG is considered to be.

## 3. Results

For the selection of candidate genes in the HKGs database, screening was conducted in PubMed. A search for the key MeSH term “housekeeping gene” (filters applied: humans) yielded 26,399 publications since 2000. A search for the keyword “housekeeping PCR” (filters applied: humans) returned 880 results since 2000. Searching for the keyword “housekeeping real-time PCR” (filters applied: humans) returned 562 results since 2000. The search term “housekeeping real-time PCR brain” (filters applied: humans) returned only 29 results since 2001. Of these 29 studies, only 4 were directly related to the analysis of HKGs in nontumor samples [22,23,24,25].

In addition, data on the analysis of reference genes in brain tissues [26], a neuronal line differentiated from bone mesenchymal stem cells [27], and human tissues [28] were used to select potential HKGs. Using the data provided in the selected seven works, a list consisting of 542 potential HKGs was compiled for further analysis.

Next, we carried out an additional screening of publications on the query “gene expression Parkinson’s disease RNA” (filter: human) in the PubMed database, and 715 publications were identified. The Section 2 for 100 publications was randomly analyzed and a list of reference genes was compiled for use in gene expression studies.

Then, we compared the two generated lists and selected the genes included in both lists to study the stability of their expression in our work. As a result of the analysis, HKGs *TBP* [25,26], *ACTB*, *RPL30* [25,29], *GAPDH* [23,29], *YWHAZ* [23], *PPIA* [27], *B2M* [26], *HPRT* [22,27], *GUSB* [25,27], *SARS1*, *AARS1* [28], *PSMD6*, *PSMD7* [26,28,29], *POLR2F* [30], *POLR2A*, and *PSMA5* [28,31] were selected.

All potential HKGs must satisfy certain conditions. First, the genome should not contain pseudogenes of these genes. It is known that processed pseudogenes lacking introns and being almost complete generate copies of mature mRNA of the corresponding genes making up 70% of all pseudogenes [32]. In this regard, one cannot be sure that the selected primer systems will be absolutely specific to the target mRNA. Based on this criterion, HKGs *ACTB* [33], *GUSB* [34], *PPIA* [35], *YWHAZ* [36], *RPL30* [37], and *GAPDH* [38] were excluded.

Second, a reference gene means a gene whose expression does not change under the conditions of the studied pathology and is also approximately the same in all studied tissues. In this regard, expression of all selected HKGs was analyzed using BioGPS, and HKG *B2M* [39] was excluded from further analysis (the difference in expression between whole brain and whole blood is more than 12-fold).

For the remaining HKGs *POLR2F*, *POLR2A*, *PSMD6*, *PSMD7*, *HPRT1*, *PSMA5*, *SARS1*, *AARS1*, *TBP*, and *BCAT2* (the names are given in the nomenclature for humans), TaqMan primer systems were selected, and an analysis of changes in their relative mRNA levels was performed in brains of mice and mouse and human blood samples. It should be noted that at the stage of selection of primer systems and their validation for analysis, the *TBP*, *POLR2F*, and *BCAT2* genes in humans were eliminated because their levels of transcripts were below the limit of detection. After obtaining the primary data, the Ct genes were analyzed using the RefFinder program.

**Figure 1:**
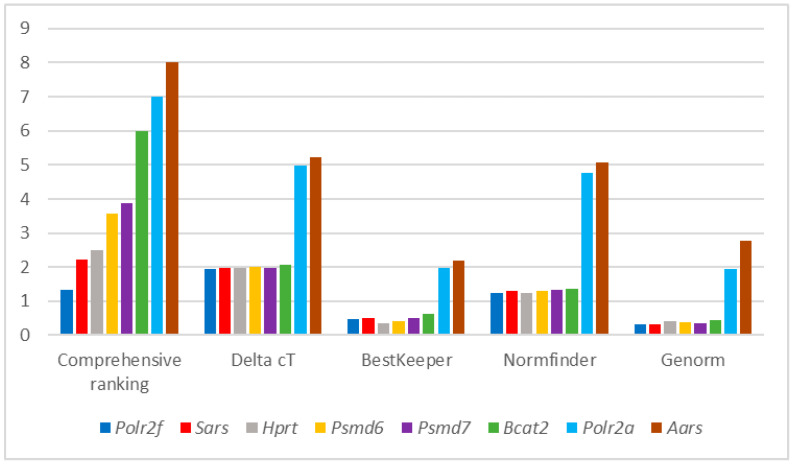
Comprehensive ranking of HKGs in mouse peripheral blood by RefFinder.

An example of data processing for the analysis of relative mRNA levels in mouse peripheral blood is shown in general in Figure 1.

The cumulative results of the analysis are shown in Table 1 and Table 2 for mice and humans. To identify the most stably expressed HKGs under PD conditions, we analyzed the stability of expression of HKGs separately in the group of control mice and healthy individuals (Table 1 and separately in the group of mice with the MPTP-induced PD model and in patients with PD (Table 2). Stability of expression was assessed based on a comprehensive score for each gene in each tissue. In each specific case (tissue and presence/absence of pathology), the most stably expressed HKG is the one with a minimum comprehensive score. For example, *Psmd7* is the most optimal and stably expressed HKG in the brain as a whole, as well as in three separate brain tissues (cerebellum, striatum, substantia nigra) in the control group of mice; however, this gene is the second in the cortex, where *Aars1* is in the first place. At the same time, *Psmd6* is the most stably expressed in the brain, as a whole, of experimental animals with the MPTP-induced PD model; however, it occupies only fourth place in the cortex and striatum and third place in the cerebellum and substantia nigra.

In general, as can be seen from the data presented, the stability of expression of the HKGs studied differs depending on the presence or absence of pathological processes.

Comprehensive scores and gene ranks were obtained for each gene in each tissue examined.

Based on the fact that the stability in different tissues and in the presence and absence of pathology is different, when carrying out work on expression analysis, it is necessary to use HKGs whose expression is most stable in a certain tissue both in the presence and in the absence of pathology. Accordingly, a table was compiled for the final ranking of HKGs, based on the calculation of the summarized comprehensive score (Table 3).

The summarized comprehensive score of each HKG in each tissue was summarized from the comprehensive score obtained from the control mice and healthy volunteers (Table 1) and mice with Parkinson-like phenotype and patients with PD (Table 2).

For example, in the brain as a whole, regardless of the presence or absence of pathology, *Aars1* is the most stably expressed and takes the first place (gene rank = 1) with a total comprehensive score of 4.21 = 2.21 + 2.20, where 2.21 is a comprehensive score from the column “whole brain” of Table 1 and 2.20 is a comprehensive score from the “whole brain” column of Table 2. Thus, the gene with the lowest value is considered the most stably expressed and takes the highest place (gene rank = 1).

The final rank of each HKG in tissues of mice was summarized from the comprehensive score obtained from the control group (Table 1) and the experimental group (Table 2). Similarly, the final rank for HKGs in human tissues was obtained. At the same time, the gene with the lowest value is considered the most stably expressed and takes the highest place (final rank).

In general, based on the data obtained, the HKG *Aars* has the most stable expression among all HKGs studied in the brain. The same gene ranks second in terms of stability of expression in various parts of the brain. Ranking second in the whole brain, *Psmd7* was most stably expressed in the striatum and substantia nigra. In the cortex, the most stably expressed HKG was *Polr2f*, and in the cerebellum, the most stably expressed HKG was *Psmd6*. It is noteworthy that the first of them was also the most stably expressed in the peripheral blood of mice, and the second was the most stably expressed in the peripheral blood of humans.

Summarized comprehensive scores and gene ranks were obtained for each gene in each tissue studied.

According to the widespread opinion of the scientific community that when analyzing changes in gene expression, it is necessary to take into account at least two HKGs, one should take into account the genes located at the first, second, and, at the most, at the third place in the stability rating of HKGs given in Table 3.

Furthermore, as the analysis shows, HKGs in similar tissues of the studied organisms are different. For example. *Polr2f* is the most stably expressed HKG in the peripheral blood of mice, whereas *PSMD6* is the most stably expressed gene in humans (Table 3).

## 4. Discussion

Currently, TaqMan qPCR is one of the most widely used methods in molecular genetics. At the same time, one of the key factors in the success of this method is the correct choice of HKGs. Today, hundreds of reference genes are known both in the literature and in commercial panels. At the same time, data from one expression study often do not reproduce the results of other similar studies; differences in the results obtained may be partly explained by the choice and instability of expression of HKGs in varying experimental conditions. A striking example is the comparison of the results of two well-performed studies. In an analysis of the stability of HKGs during the reprogramming of the mouse neural stem cell line N31, Panina and coauthors in 2018 showed that *GAPDH* is one of the most stably expressed HKGs among those analyzed [40]. However, in 2020, the same group of scientists, when analyzing HKGs in induced pluripotent stem cells of the same lineage, concluded that *GAPDH* has a highly variable expression during reprogramming. This gene is not attractive as a reference gene for the analysis of expression in works related to the process of fibroblast reprogramming [41]. The authors point out that the way the primers were selected changed from the first publication to the second. With this in mind, they explain that in the first of these works, the *Rps18* gene was considered as one of the variable genes while in the second work, the authors recognized *Rps18* as a stably expressed HKG.

In this work, we analyzed the relative mRNA levels of reference genes in mouse brain tissues, as well as in the peripheral blood of mice and humans. The analysis was carried out in samples of brain tissues and blood of mice with modeling of Parkinson-like phenotype and in matching samples from a control group of mice. The work presented here was aimed, first of all, at studying the stability of expression of HKGs in the main tissues of the mouse brain, as one of the main objects of PD modeling. At the same time, a toxic model based on the introduction of MPTP (1-methyl-4-phenyl-1,2,3,6-tetrahydropyridine) was chosen. Models based on the introduction of this toxin are currently considered classical [42]. It has been shown that MPTP has an affinity for the DAT dopamine transporter, which causes a key feature of PD, degeneration of dopaminergic neurons and dopamine deficiency in the striatum [43,44]. For the analysis, we used a model of the early symptomatic stage of PD based on the administration of MPTP, similar to stages 1–2 on the Hoehn–Yahr scale in patients with PD [45]. In addition, peripheral blood samples from patients with PD (stages 1–2 on the Hoehn–Yahr scale), as well as healthy volunteers, were studied.

Based on a bioinformatic analysis searching for the presence of pseudogenes, the genes *ACTB*, *GUSB*, *PPIA*, *YWHAZ*, and *GAPDH*, widely used as HKGs, were removed from further study. For example, *ACTB* and *GAPDH* are the most frequently used housekeeping systems. *ACTB* has 22 pseudogenes and *GAPDH* has 47 [46]. Pseudogenes can be amplified together with cDNA obtained from the target RNA. On the one hand, treatment with DNases can serve as a solution to this problem, although this greatly decreases the yield of isolated RNA. This method is of little use considering that the purpose of the study was to analyze changes in the levels of poorly presented transcripts. On the other hand, amplification of genomic DNA can be excluded by selecting primers for exon–exon junctions, and thus only the target cDNA obtained from mature RNA devoid of introns will be expressed. As noted above, it is known that processed pseudogenes lack introns, are almost complete copies of mature mRNAs of the corresponding genes, and make up 70% of all pseudogenes [32,46]. In this regard, one cannot be sure that the selected primer system will be absolutely specific for the target mRNA, and it is appropriate not to select genes having pseudogenes for use as HKGs [47]. 

The rest of the HKGs selected based on the results of the literature analysis passed the bioinformatic analysis. Coincidentally, in silico pseudogenes were found for the *PSMD7* (NM_002811.5) and *HPRT1* (NM_000194.3) genes. However, there is currently no experimental confirmation of their existence, so we selected these genes for further analysis. In addition, primers were selected so that they did not anneal to the region of putative pseudogenes in the genome.

One of the fundamentally important conditions in the selection of HKGs is the stability of their expression level, regardless of the presence or absence of the development of a pathological process. In our work, it was shown that the presence of a pathological process affects the stability of the expression of HKGs. As can be seen from Table 1 and Table 2, none of the HKGs with the lowest score and, correspondingly, the highest rank in the control group of mice (Table 1) coincide with those in the group of mice with MPTP-induced PD (Table 2). A similar situation is observed when analyzing HKGs in human peripheral blood.

Overall, the most stably expressed HKG in the mouse brain (Table 3) is *Aars*, which encodes an alanyl-tRNA synthetase. This gene is not directly involved in the processes associated with the functioning of the nervous system and processes involved in the pathogenesis of PD. In addition, this gene is the second most stably expressed gene in some of the brain tissues we studied. Thus, this gene is the most stably expressed and attractive HKG for expression analysis.

The second most stably expressed gene in the mouse brain is the gene associated with the functioning of proteasome subunits. Even though the processes associated with the functioning of ubiquitin-dependent proteolysis are involved in the pathogenesis of PD, the *Psmd7* gene in mice turned out to be one of the most stably expressed in the brain as a whole (Table 3). The same gene is the most stably expressed in the substantia nigra and striatum.

It should be noted that transcriptome studies in the human brain are possible only in postmortem tissues. Unfortunately, such studies do not reflect the processes involved in the pathogenesis of the disease, especially in the early stages. In this regard, the optimal approach to research in vivo is the analysis of peripheral blood that allows studies of the pathogenesis of the disease, including its early stages.

The gene encoding polymerase 2 subunits in mice, *Polr2f*, and gene encoding proteasome subunit 6 in humans, *PSMD6*, have the least variability in their expression when analyzing peripheral blood. These data are not surprising because the functioning of the polymerase in the cell is of decisive importance for its vital activity. In this regard, the stability of expression of *Polr2f* as HKGs is beyond doubt. Based on all the data obtained, it can be concluded that when analyzing changes in gene expression under pathological conditions, it is necessary to select HKGs for each specific tissue under study. As shown in Table 3 for the mouse brain, even if the stability of the relative mRNA levels of some reference gene, for example, *Aars*, is shown for the organ as a whole, this does not mean that it will be the most effective HKG for other tissues, which is shown, for example, for the cerebellum (*Psmd6*).

## 5. Conclusions

The data obtained indicate that there is no universal HKG both for different brain tissues of one organism and for similar tissues of different organisms. This conclusion is supported by other studies: researchers must select and validate a set of reference genes for each specific cell line [41]. 

In addition, we have shown that the presence of a pathological process may affect the expression of HKGs. This fact must be taken into account when choosing the optimal HKG for expression analysis in pathological conditions. In this case, it is necessary to choose those HKGs that are most stably expressed in both experimental and control groups, in other words, regardless of experimental conditions.

In conclusion, it should be noted that there are certain limitations of the use of the HKGs selected in this study as the most stably expressed. Thus, these HKGs can be most confidently used to study the pathological process caused by the pathogenesis of PD in young mice, as well as for PD patients at relatively early stages of the pathological process.

## Figures and Tables

**Table 1:** Table of the stability of expression of potential HKGs in the brain and peripheral blood of control group of mice and peripheral blood of control group of mice and healthy volunteers according to the RefFinder program.

Gene Rank ^2^	Mice												Humans	
	Whole Brain		Cortex		Cerebellum		Striatum		Substantia Nigra		Blood		Peripheral Blood	
	HKG	Score ^1^	HKG	Score	HKG	Score	HKG	Score	HKG	Score	HKG	Score	HKG	Score
1	*Psmd7*	1.57	*Aars*	1.68	*Psmd7*	1.86	*Psmd7*	1.00	*Psmd7*	1.32	*Sars*	1.57	*SARS1*	1.57
2	*Aars*	2.21	*Psmd7*	2.06	*Psmd6*	2.63	*Aars*	2.21	*Aars*	1.68	*Polr2f*	2.38	*PSMD6*	2.11
3	*Polr2a*	2.45	*Polr2f*	2.21	*Hprt*	3.13	*Hprt*	3.31	*Polr2a*	2.83	*Polr2a*	2.71	*HPRT1*	3.16
4	*Bcat2*	2.99	*Psmd6*	3.83	*Aars*	3.31	*Polr2a*	3.31	*Bcat2*	3.87	*Hprt*	3.98	*POLR2A*	3.22
5	*Psmd6*	3.98	*Bcat2*	4.53	*Polr2f*	4.30	*Sars*	4.56	*Sars*	4.61	*Psmd7*	4.16	*PSMA5*	3.94
6	*Hprt*	6.24	*Hprt*	4.95	*Bcat2*	4.79	*Bcat2*	5.92	*Polr2f*	6.09	*Psmd6*	4.92	*PSMD7*	4.56
7	*Sars*	7.24	*Polr2a*	6.44	*Polr2a*	5.66	*Psmd6*	6.48	*Psmd6*	6.24	*Bcat2*	5.14	*AARS1*	7.00
8	*Polr2f*	7.44	*Sars*	8.00	*Sars*	5.73	*Polr2f*	8.00	*Hprt*	8.00	*Aars*	8.00		

^1^ Score—a comprehensive score calculated using the RefFinder program. ^2^ Gene rank (a place in the rating of stability of expression) is based on sorting HKGs by their comprehensive scores from minimum to maximum. The first place in the ranking (gene rank = 1) is assigned to the gene with the minimum comprehensive score, and this gene is considered the most stably expressed HKG in the tissue under study. The last place in the ranking (gene rank = 8) is assigned to the gene with the maximum comprehensive score, and this HKG is considered the least stably expressed HKG.

**Table 2:** Table of the stability of expression of potential HKGs in the brain and peripheral blood of experimental group of mice and peripheral blood of experimental group of mice and PD patients according to the RefFinder program.

Gene Rank ^2^	Mice												Humans	
	Whole Brain		Cortex		Cerebellum		Striatum		Substantia Nigra		Blood		Peripheral Blood	
	HKG	Score ^1^	HKG	Score	HKG	Score	HKG	Score	HKG	Score	HKG	Score	HKG	Score
1	*Psmd6*	1.86	*Bcat2*	1.32	*Aars*	1.78	*Sars*	1.50	*Hprt*	1.50	*Hprt*	1.78	*POLR2A*	1.41
2	*Aars*	2.00	*Polr2f*	2.06	*Polr2f*	1.86	*Psmd7*	2.21	*Psmd7*	2.00	*Polr2f*	1.86	*PSMD6*	2.45
3	*Polr2f*	2.11	*Aars*	2.63	*Psmd6*	2.45	*Aars*	3.25	*Bcat2*	3.00	*Psmd6*	2.45	*PSMA5*	2.82
4	*Psmd7*	3.08	*Psmd6*	3.46	*Polr2a*	2.63	*Polr2a*	3.46	*Psmd6*	3.83	*Psmd7*	2.83	*PSMD7*	3.98
5	*Sars*	4.16	*Polr2a*	5.00	*Bcat2*	5.38	*Bcat2*	4.60	*Aars*	4.43	*Sars*	4.61	*SARS*	4.23
6	*Bcat2*	6.24	*Psmd7*	5.09	*Psmd7*	5.48	*Hprt*	4.61	*Polr2f*	5.12	*Bcat2*	5.73	*HPRT1*	4.30
7	*Polr2a*	7.24	*Sars*	7.24	*Sars*	6.96	*Psmd6*	5.96	*Sars*	5.44	*Polr2a*	7.00	*AARS1*	6.00
8	*Hprt*	7.44	*Hprt*	7.44	*Hprt*	7.74	*Polr2f*	7.20	*Polr2a*	8.00	*Aars*	8.00		

^1^ Score—a comprehensive score calculated using the RefFinder program. ^2^ Gene rank (a place in the rating of stability of expression) is based on sorting HKGs by their comprehensive scores from minimum to maximum. The first place in the ranking (gene rank = 1) is assigned to the gene with the minimum comprehensive score, and this gene is considered the most stably expressed HKG in the tissue under study. The last place in the ranking (gene rank = 8) is assigned to the gene with the maximum comprehensive score, and this HKG is considered the least stably expressed HKG.

**Table 3:** Summary table of the stability of expression of potential HKGs based on total score in the brain and peripheral blood of mice and human peripheral blood according to the RefFinder program regardless of the presence or absence of pathology.

Gene Rank ^2^	Mice												Humans	
	Whole Brain		Cortex		Cerebellum		Striatum		Substantia Nigra		Peripheral Blood		Peripheral Blood	
	HKG	S-Score ^1^	HKG	S-Score	HKG	S-Score	HKG	S-Score	HKG	S-Score	HKG	S-Score	HKG	S-Score
1	*Aars*	4.21	*Polr2f*	4.27	*Psmd6*	5.08	*Psmd7*	3.21	*Psmd7*	3.32	*Polr2f*	4.24	*PSMD6*	4.56
2	*Psmd7*	4.65	*Aars*	4.31	*Aars*	5.09	*Aars*	5.46	*Aars*	6.11	*Hprt*	5.76	*POLR2A*	4.63
3	*Psmd6*	5.84	*Bcat2*	5.85	*Polr2f*	6.16	*Sars*	6.06	*Bcat2*	6.87	*Sars*	6.18	*SARS1*	5.80
4	*Bcat2*	9.23	*Psmd7*	7.15	*Psmd7*	7.34	*Polr2a*	6.77	*Hprt*	9.50	*Psmd7*	6.99	*PSMA5*	6.76
5	*Polr2f*	9.55	*Psmd6*	7.29	*Polr2a*	8.29	*Hprt*	7.92	*Sars*	10.05	*Psmd6*	7.37	*HPRT1*	7.46
6	*Polr2a*	9.69	*Polr2a*	11.44	*Bcat2*	10.17	*Bcat2*	10.52	*Psmd6*	10.07	*Polr2a*	9.71	*PSMD7*	8.54
7	*Sars*	11.40	*Hprt*	12.39	*Hprt*	10.87	*Psmd6*	12.44	*Polr2a*	10.83	*Bcat2*	10.87	*AARS1*	13.00
8	*Hprt*	13.68	*Sars*	15.24	*Sars*	12.69	*Polr2f*	15.20	*Polr2f*	11.21	*Aars*	16.00		

^1^ S-Score—the summarized comprehensive score for each HKG in each tissue was calculated as the sum of the scores from Table 1 and Table 2. ^2^ Gene rank is based on sorting HKGs by their summarized comprehensive score from the minimum value, where the corresponding gene is assigned gene rank = 1, to the maximum value, where the corresponding gene is assigned gene rank = 8. The 1st place in the ranking (gene rank = 1) belongs to the most stable housekeeping gene.

## Data Availability

Not applicable.

## Supplementary Materials

The following are available online at https://www.mdpi.com/article/10.3390/cells10092252/s1, Table S1: Sequences of gene-specific primers and probes for qPCR (TaqMan).
