# Supplementary material for: Housekeeping Genes for Parkinson’s Disease in Humans and Mice
Source: Cells. 2021 Aug 30;10(9):2252. doi: 10.3390/cells10092252 (PMC8470043; doi:10.3390/cells10092252)
Supplement: Supplementary file 1 [file cells-10-02252-s001.zip › cells-1328722-supplementary.pdf]

Supplement S1. Sequences of gene-specific primers and probes foк qPCR (TaqMan)

| Gene | Nucleotide sequences |
|---|---|
| *AARS1*<br>NM_001605.2 | Probe: 5'-FAM-TATGTTCACTCGTCTGCCACCATCCCAT-BHQ1-3'<br>Forward primer: 5'- CGGCAGCGATTTATAGATTTCTTCAAG-3'<br>Reverse primer: 5'- GGTGAGATGGGTCAATTGTGTTCA-3' |
| *BCAT2*<br>*NM_001190.4* | Probe: 5'- FAM- CCTACTTCCCTGGAGGCTCCG-BHQ1-3'<br>Forward primer: 5'- CGCTCCTGTTCGTCATTCTCT-3'<br>Reverse primer: 5'- GGGCCCATAATTCCCACCTAAC-3' |
| *HPRT1*<br>NM_000194.2 | Probe: 5'-FAM- TGGCCTCCCATCTCCTTCATCACATCTCGA -BHQ1-3'<br>Forward primer: 5'- TCATTATGCTGAGGATTTGGAAAGGG -3'<br>Reverse primer: 5'- GCACACAGAGGGCTACAATGTG -3' |
| *POLR2A*<br>NM_000937.4 | Probe: 5'-FAM-CCACCTGGTTGATGGAGTTCCGCACAGTC-BHQ1-3'<br>Forward primer: 5'-AGACTGCTGAGACTGGATACATCC-3'<br>Reverse primer: 5'-AGGCCGTCTTCGCCGTAG-3' |
| *POLR2F*<br>NM_021974.3 | Probe: 5'-FAM-CTTCATCCTCCTCCACATCATCAAAGTCGTCG-BHQ1-3'<br>Forward primer: 5'-ATGTCAGACAACGAGGACAATTTTG-3'<br>Reverse primer: 5'-TCTTCGGCATTCTCCAAGTCATC-3' |
| *PSMA5*<br>NM_001199772.1 | Probe: 5'-FAM-AGCCATCAAGTCTTCACTCATCATCCTC-BHQ1-3'<br>Forward primer: 5'-AGAAGTTTACCACAAGTCTATGAC-3'<br>Reverse primer: 5'-CATTCAGCTTCTCCTCCATTAC-3' |
| *PSMD6*<br>NM_001271779.1 | Probe: 5'-FAM- AGGCGGTTTCTCCTGTCCCAGTCTCCTC -BHQ1-3'<br>Forward primer: 5'- AACACAGAAAAGGCCAAAAGCTTAAT -3'<br>Reverse primer: 5'- AATAGCCACACAATAAAGACCCTGAT -3' |
| *PSMD7*<br>NM_002811.4 | Probe: 5'-FAM-GCTTCTGTAGGCAGCCCTAGGTCCTTCGG-BHQ1-3'<br>Forward primer: 5'-TGTCCTAATTCCGTATTGGTCATCATTG-3'<br>Reverse primer: 5'-CGTGTTCAAATGTTTTCGAGGTTGG-3' |
| *SARS1*<br>NM_001330669.1 | Probe: 5'-FAM-TCGCCACTCGCTGTCTGCCTTCACCA-BHQ1-3'<br>Forward primer: 5'-CCCAGCCCTCATCCGAGAG-3'<br>Reverse primer: 5'-TGTTCAAGTTGTCTGCCCGAAATC-3' |
| *TBP*<br>NM_003194.5 | Probe: 5'- FAM- GAAGGCCTTGTGCTCACCCACCAACAATT-BHQ1-3'<br>Forward primer: 5'- GGAGCTGTGATGTGAAGTTTCCTA-3'<br>Reverse primer: 5'- GGAGAACAATTCTGGGTTTGATCATT-3' |
| *Aars*<br>NM_146217.4 | Probe: 5'- FAM-ACCCCACTCTGCTCTTCGCCAACG-BHQ1-3'<br>Forward primer: 5'-TCCACTCTTCCGCCACCAT-3'<br>Reverse primer: 5'-CTGCTCAGCTTCGCCATAGG-3' |
| *Bcat2*<br>*NM_001243053.1* | Probe: 5'-FAM-CGGATACACTCCAACAGCTCCTGCTTGT-BHQ1-3'<br>Forward primer: 5'-TCAACATGGACAGGATGCTACG-3'<br>Reverse primer: 5'-CCAGTCTTTGTCTACTTCAATGAGC-3' |
| *Hprt*<br>NM_013556.2 | Probe: 5'- FAM- CCTGTATCCAACACTTCGAGAGGTCCTT- BHQ1-3'<br>Forward primer: 5'-ACAGCCCCAAAATGGTTAAGGT-3'<br>Reverse primer: 5'-CCAACAACAAACTTGTCTGGAATTTC-3' |
| *Polr2a*<br>*NM_001291068.1* | Probe: 5'- FAM-TCACAGACATCCGCTTCAATTCATCCG- BHQ1-3'<br>Forward primer: 5'- CACCATCAAGAGAGTGCAGTTC-3'<br>Reverse primer: 5'- CCTCCGTTGTTTCTGGGTATTTG-3' |
| *Polr2f*<br>*NM_027231.2* | Probe: 5'- VIC-CCTCAGCATTTTCCAAGTCGTCAAGTCC-BHQ2-3'<br>Forward primer: 5'- TGTCAGACAACGAGGACAATTTCG-3'<br>Reverse primer: 5'- TCGCTCACCAGATGGGAGAATC-3' |
| *Psma5*<br>*NM_011967.3* | Probe: 5'- FAM- GGTCCGAGTACGACAGGGGTGTGAATACT-3'<br>Forward primer: 5'- GGTTGATTAGGGTGCTGCTTTC-3'<br>Reverse primer: 5'- TCCACTTGAAATAATCTTCCTTCAGG-3' |
| *Psmd6*<br>NM_025550.2 | Probe: 5'- FAM-AATCGTGGAGACCAACAGACCTGATAGCAA- BHQ1-3'<br>Forward primer: 5'-GGTGTGGGTGTGGACTTCATT-3'<br>Reverse primer: 5'-CTCCTTTCTTGATGGTTTCTTGATACTG-3' |
| *Psmd7*<br>*NM_010817.2* | Probe: 5'-FAM-AGTCCTAGGTCCTTTGGCTTCACGTCGA-BHQ1-3'<br>Forward primer: 5'-CTGCACAAGAATGATATCGCCATC-3'<br>Reverse primer: 5'-CTCCACTGAGATGTAGGCTTCG-3' |

| | |
|---|---|
| *Sars*<br>*NM_011319.3* | Probe: 5'- FAM- CGTTCTACTTTGTTGTCTGCGTCCTCATCA- BHQ1-3'<br>Forward primer: 5'-GCGAGATTGGGAACCTTCTG-3'<br>Reverse primer: 5'-ATGGGAATACTTCTTCCTGACTGTA-3' |
| *Tbp*<br>*NM_013684.3* | Probe: 5'- FAM-CACTCCTGCCACACCAGCTTCTGAGAGC-BHQ1-3'<br>Forward primer: 5'-CCTTCACCAATGACTCCTATGACC-3'<br>Reverse primer: 5'-CAGCCAAGATTCACGGTAGATACA-3' |

*accession numbers in GenBank database;
**FAM,** fluorescent dye, **BHQ1,** fluorescent quencher.
